# Exploring the Genetic Diversity of Epstein–Barr Virus among Patients with Gastric Cancer in Southern Chile

**DOI:** 10.3390/ijms241411276

**Published:** 2023-07-10

**Authors:** María Elena Reyes, Louise Zanella, Ismael Riquelme, Kurt Buchegger, Bárbara Mora-Lagos, Pablo Guzmán, Patricia García, Juan C. Roa, Carmen Gloria Ili, Priscilla Brebi

**Affiliations:** 1 Instituto de Ciencias Biomédicas, Facultad de Ciencias de la Salud, Universidad Autónoma de Chile, Temuco 4810101, Chile; maria.reyes@uautonoma.cl (M.E.R.); ismael.riquelme@uautonoma.cl (I.R.); barbara.mora@uautonoma.cl (B.M.-L.); 2 Doctorado en Ciencias Médicas, Universidad de La Frontera, Temuco 4811230, Chile; louise.zanella@ufrontera.cl; 3 Núcleo Milenio de Sociomedicina, Santiago 7560908, Chile; 4 Laboratory of Integrative Biology (LIBi), Millennium Institute on Immunology and Immunotherapy, Center of Excellence in Translational Medicine-Scientific and Technological Bioresource Nucleus-(-CEMT-BIOREN), Universidad de La Frontera, Temuco 4810296, Chile; kurt.buchegger@ufrontera.cl; 5 Departamento de Ciencias Básicas, Facultad de Medicina, Universidad de La Frontera, Temuco 4811322, Chile; 6 Pathology Department, School of Medicine, Universidad de La Frontera, Temuco 4781176, Chile; pablo.guzman@ufrontera.cl; 7 Millennium Institute on Immunology and Immunotherapy, Department of Pathology, School of Medicine, Pontificia Universidad Católica de Chile, Santiago 8330024, Chile; pgarciam@uc.cl (P.G.); jcroa@med.puc.cl (J.C.R.)

**Keywords:** gastric cancer, Epstein–Barr virus (EBV), *EBNA3A*, phylogeny, Chile

## Abstract

The Epstein–Barr virus (EBV) has been associated with gastric cancer (GC), one of the deadliest malignancies in Chile and the world. Little is known about Chilean EBV strains. This study aims to investigate the frequency and genetic diversity of EBV in GC in patients in southern Chile. To evaluate the prevalence of EBV in GC patients from the Chilean population, we studied 54 GC samples using the gold standard detection method of EBV-encoded small RNA (EBER). The EBV-positive samples were subjected to amplification and sequencing of the Epstein–Barr virus nuclear protein 3A (*EBNA3A*) gene to evaluate the genetic diversity of EBV strains circulating in southern Chile. In total, 22.2% of the GC samples were EBV-positive and significantly associated with diffuse-type histology (*p* = 0.003). Phylogenetic analyses identified EBV-1 and EBV-2 in the GC samples, showing genetic diversity among Chilean isolates. This work provides important information for an epidemiological follow-up of the different EBV subtypes that may cause GC in southern Chile.

## 1. Introduction

Gastric cancer (GC) is the sixth most common cancer type, with an incidence of 11.1 cases per 100,000 people, and the fifth most deadly malignancy globally, with a mortality rate of 7.7 per 100,000 people [1]. GC is mainly classified based on the histopathological features observed in tumors as either intestinal (I) or diffuse (D). The intestinal type is distinguished by the presence of cohesive cells that are organized into gland-like structures. By contrast, the diffuse type is characterized by tumor cells that do not interact with each other and instead infiltrate the surrounding tissue as individual cells or small clusters [2]. The average frequency ratio (I/D) is 1.3 [2,3].

In 2014, The Cancer Genome Atlas (TCGA) research network analyzed 295 stomach tumors, which led to the identification of four major genomic subtypes of GC: genomically stable (GS), microsatellite instability (MSI), chromosomal-instable (CIN), and Epstein–Barr virus(EBV)-positive [4,5]. The EBV-positive subgroup showed distinctive molecular characteristics, including, but not limited to, the highest hypermethylation rates in the host DNA [6] compared to other cancers, frequent mutations in the *PIK3CA*, *JAK2*, and *CD274 (PD-L1)* genes [7], and deletions in key tumor suppressor genes. The EBV-positive subtype represented 8.8% of the total cohort of TCGA [4].

EBV is a double-stranded DNA virus ~170 Kb in length [8]. EBV has been linked with different diseases, including infectious mononucleosis [9], post-transplant lymphoproliferative disease [10], multiple sclerosis [11], systemic lupus erythematosus [12], and several cancers. In EBV-associated cancer, Burkitt lymphoma (BL) [13], Hodgkin’s lymphoma (HL) [14], leiomyosarcomas in HIV-positive children [15], lymphomas of T/NK cell origin (LTNK) (aggressive NK leukemia) [16], nasopharyngeal carcinoma (NPC) [17], and GC are commonly found. The incidence range of primary cancers associated with EBV infection (2020) in descending order is NPC (105,500/120,600), GC (82,800/116,400), HL (34,300/52,400), LTNK (5500/34,700), and BL (6600) [18].

This virus is classically classified into two genotypes, EBV-1 (or A) and EBV-2 (or B), using specific polymorphisms of the EBV nucleotide antigen 2(EBNA2) and EBNA3A, 3B, and 3C viral genes [19]. EBV nuclear antigens (EBNAs) are proteins expressed during latent infection and impact the transcription of the virus and host cell. In the case of GC, a latent infection is often present, where EBV infects host cells and remains dormant without active replication [20]. However, the activation of EBV latency genes can lead to significant abnormalities in the host genome, particularly concerning atypical DNA methylation [21].

A previous study provided a more comprehensive classification based on the whole viral genome, differentiating up to 12 EBV phylopopulations [22]. These findings suggest that diversity in EBV populations may contribute to a specific phenotypical disease. In fact, EBV sequence variations have been proposed to be related to the phylogeographic diversity of Epstein–Barr virus-associated gastric carcinoma (EBVaGC) [23] and associated with some types of tumors [22].

The primary objective of this study was to investigate the genetic diversity of EBV in southern Chile by reconstructing the phylogenetic relationship between EBV isolates. First, we determined the prevalence of EBV within the GC bulk and its prognostic significance among GC cases in southern Chile. This research is significant because GC is the primary cause of cancer-related deaths in Chile, with a high prevalence (11.9 deaths per 100,000 people in 2018) [24], and EBV has been suggested as a relevant factor for gastric carcinogenesis in this population [25]. This is true particularly in the country’s southern region, where the highest mortality rates due to GC are concentrated, exceeding the national average with an average rate of 14 for women and 36.4 for men [26].

To this end, we used the gold standard detection method, EBV-encoded small RNA (EBER) detection through in situ hybridization (EBER-ISH) and PCR. Subsequently, we conducted a phylogenetic reconstruction based on EBNA3A gene sequencing data. Our findings indicate the presence of EBV-1 and EBV-2 in southern Chile and a significant association with diffuse-type histology.

## 2. Results

### 2.1. Cohort Description

Based on Lauren’s classification criteria, 33 cases (61.1% of the total cohort) were identified as intestinal-type gastric carcinomas, while 21 cases (38.9% of the entire cohort) were classified as diffuse-type gastric carcinomas.

The study population (*n* = 54) was predominantly male (*n* = 38; 70.4%). The age of the subjects ranged between 32 and 85 years old, with a median age of 66 years. Our findings suggest no significant association exists between age and frequency of EBV-positive GC, EBVaGC, (*p* = 0.208) among the study population. This study employs samples from a past cohort collected between 2008 and 2012.

### 2.2. Frequency of EBV and Its Association with Clinicopathological Features

**Figure 1:**
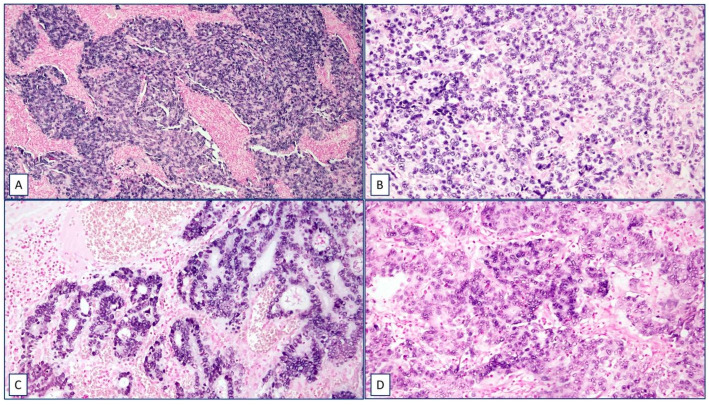
Representative cases of EBV nuclear positivity in gastric carcinomas detected using EBER-ISH in FFPE tissues. (**A**) Syncytial pattern with high inflammatory infiltration 10×; (**B**,**D**) low intermixed inflammatory infiltration among tumoral cells 20×; (**C**) tubular pattern and moderate lymphocyte infiltration 20×.

**Figure 2:**
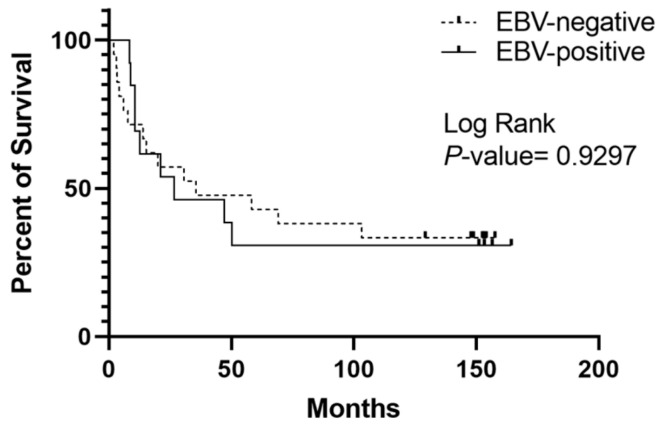
Kaplan–Meier curve for the cumulative survival rate of patients with gastric tumors using the EBER-ISH technique. Interestingly, these values and survival curve are similar to what would obtained based on EBV positivity using PCR. The solid line indicates patients whose tumors were positive for EBV, and the dashed line indicates patients with tumors negative for EBV (*p* = 0.9297, log-rank test).

An EBER-ISH analysis was conducted on FFPE GC tissues, revealing that 20% (*n* = 11) of samples were EBV-EBER-positive. Most EBV-EBER-positive samples exhibited a nuclear staining pattern in their cells, as shown in Figure 1. Only one sample displayed a combined nuclear and cytoplasmic staining pattern. Furthermore, a survival analysis revealed no significant correlation between the presence of EBV in GC tissues and poor overall survival (*p* = 0.9297; Figure 2; Table 1).

PCR analysis was performed on DNA extracted from fresh-frozen GC tissues to examine the EBNA3A gene, revealing that 12 (22%) samples tested positive for EBV. Among these, 11 samples were from males, which accounted for 20.4% of the total cohort and 91.7% of the total EBV-positive samples. Conversely, only one female sample (1.85% of the entire cohort) was positive for EBV, representing 8.3% of the total EBV-positive samples. These findings suggest a potential association between males and a higher frequency of EBV-positive GC (*p* = 0.064; Table 2).

Regarding Lauren’s classification, out of the total cohort, only three intestinal-type samples were positive for EBV, which accounts for 16.7% of the total cohort and 25% of EBV-positive samples. Of the diffuse-type histology samples, nine were positive for EBV, accounting for 5.6% of the total cohort and 75% of EBV-positive samples. This result suggests a significant association between diffuse-type histology and EBV positivity (*p* = 0.003, Table 2).

However, no associations were found between EBV status and patient ethnicity (Hispanic or Amerindian), anatomical location, differentiation degree, or TNM tumor staging. The distribution of EBVaGC cases according to other clinicopathological features is also summarized in Table 2.

### 2.3. Phylogenetic Classification of the EBV Strains

**Figure 3:**
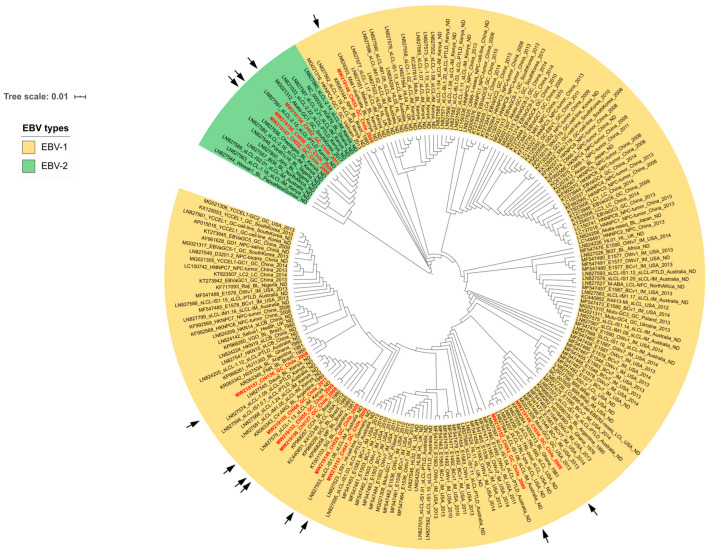
Phylogenetic tree sorting the 12 EBV isolates from Chile according to EBV-1 and 2 classifications. Maximum likelihood (ML) tree based on the terminal sequence of EBNA3A gene (1860 pb), including the 188 isolates available in the public database and the 12 Chilean EBV isolates (red letters) obtained from samples. Black arrows indicate the position of the isolates in the tree. Those isolates classified as EBV-1 are in yellow, and those classified as EBV-2 are in green. The bootstrap value (1000 replicates) is shown on the branch.

In this study, 12 EBVaGC samples from the Chilean population (CHI isolates) were screened and sequenced to determine the presence of EBV isolates. Phylogenetic tree reconstruction based on the terminal region of the EBNA3A gene of the virus in conjunction with 188 EBV sequences available in online databases made it possible to classify all isolates into EBV-1 and EBV-2. Most of the EBV isolates from the Chilean cohort (CHI29, CHI33, CHI34, CHI47, CHI53, CHI55, CHI66, CHI127, and CHI4130) belong to EBV-1. However, these isolates clustered into different subclades, indicating a marked viral diversity (Figure 3, yellow clades). The EBV-1 clade is cosmopolitan, since it contains EBV sequences from different geographical origins, such as North America (USA), South America (Argentina and Brazil), Europe (Ukraine, United Kingdom, and Poland), Africa (Ghana, Kenya, and North Africa), Asia (China, Japan, Republic of Korea, and Vietnam), and Oceania (Australia). In addition, Chilean EBV isolates belonging to the EBV-1 clade showed similarities with those associated with certain diseases, such as BL, GC, PTLD, spontaneous lymphoma cell lines (Slcl), and IM.

Conversely, the remaining three Chilean isolates (CHI32, CHI39, and CHI62) were classified within the EBV-2 clade (Figure 3, green clades). Interestingly, the CHI62 isolate clustered into a different subclade from CHI32 and CHI39, indicating viral diversity inside the EBV-2 clade as well. The EBV-2 cluster mainly showed geographical association with isolates from North Africa, Ghana, Kenya, Nigeria, Australia, and Papua New Guinea.

## 3. Discussion

In 2014, the TCGA consortium published a study on GC that provides valuable insights into the role of EBV in the development of cancer, as well as the unique molecular characteristics of the EBVaGC subtype [4]. However, the involvement of EBV as a trigger for the development of GC has been described in the Chilean population [25,27,28]. Our study aimed to evaluate the frequency of EBVaGC within a set of GC samples obtained from a population in southern Chile. In light of the potential deletion of the EBNA-2 gene in specific EBV strains, as discussed previously in the literature [29], and the limited effectiveness of EBNA-2 in distinguishing all members of genotype 2 (EBV-2), we intentionally decided to amplify and sequence the EBNA3A gene. The finding of our study showed that 22.0% of GC cases were positive for EBV upon evaluating the EBNA3A gene using PCR. This prevalence is among the highest worldwide and constitutes a 2.5-fold increase in the frequency found in the TCGA study (8.8%) [4]. The presence of EBVaGC is influenced by geographical location, with prevalence rates ranging from 5% to 15% in gastric tumors that test positive for the virus. Higher rates are observed in South and Central America compared to Asia and Europe [18]. Among the genetic characteristics associated with EBV in tumors, one notable feature is the abnormal methylation of DNA [30]. EBV actively contributes to oncogenic changes by modifying the epigenetic profile of the host cell’s genome, particularly by targeting tumor suppressor genes such as E-cadherin, which is known to be epigenetically silenced in gastric carcinomas positive for the virus [18].

It is important to note that other studies around the world have reported varying prevalence rates of EBV-positive GC cases, such as 21.4% in Colombia [31], 14.0% in the United States [32], 11.1% in Japan [33], 10.5% in Brazil [34], and 4.9% in China [35]. In Chile, two studies by Corvalan et al. published in 2001 [25] and 2005 [27] reported frequencies of 16.8% and 23.6% of EBV-positive GC, respectively. Additionally, a 2020 study included 91 GC patients and evaluated EBV status, finding 13.2% EBV-positive samples [36]. All three studies mentioned above analyzed patient populations from Santiago (the capital of Chile) and performed EBV detection through EBER-ISH analysis. The prevalence of EBV found in our study was close to the second study by Corvalan et al. from 2005 [27]. There are some differences between our present study and those previously conducted in Chile. For instance, our study evaluated a population from the southern region, while previous studies analyzed populations from central Chile (including Santiago). Our detection method involved using EBER-ISH in addition PCR, whereas previous studies only used EBER-ISH alone. Moreover, it is important to mention that the central region has a more ethnically heterogeneous population than the La Araucanía region, where our study was conducted. This region has a higher concentration of immigrants (61.3%) compared to La Araucanía (0.6%) [37]. Furthermore, the Mapuche ethnicity is more prevalent in La Araucanía, representing 32.8% of the population, compared to 8.6% in the metropolitan region [38]. It has been suggested that these differences in ethnicity explain the high incidence of certain cancers, including GC and gallbladder cancer (GBC) [39]. However, our study found no significant association between ethnicity and EBV-positive GC cases.

As reported by other studies, we also observed a higher frequency of EBV-positive GC cases among male patients [40,41]. Although the results did not reach statistical significance in our study due to the sample size (*p =* 0.064), this could be a relevant factor if the sample size were increased. One hypothesis is that tumor suppressor genes on the X chromosome may contribute to the higher prevalence of GC in males than in females, but a clear explanation is yet to be determined [42,43]. Regarding age, while some studies have reported a significant association between EBVaGC and younger age [44,45], our results did not show any significant differences between age and the presence of EBV in tumors. This finding is consistent with the results of a recent meta-analysis [40].

According to Lauren’s classification, EBV-positive GC cases showed a significant association with diffuse-type GC (*p* = 0.003). Previous studies conducted either on patients in Chile or worldwide also showed a significant association with diffuse histology [25,40]. Diffuse GC is characterized by low cell cohesion, greater replacement of normal gastric mucosa with seal ring cells [46], higher gastric wall invasion without intestinal metaplasia, and fast progression [47]. In fact, diffuse-type GC has a worse prognosis than intestinal-type GC [48]. Interestingly, 25% of the EBVaGC samples were intestinal-type tumors, which are also closely related to *H. pylori* infection, showing a well-defined carcinogenic sequence. We found no statistical differences between other clinicopathological characteristics, such as cancer stage, differentiation, or anatomical location of the tumor.

The Kaplan–Meier curves of the GC cases showed no statistically significant differences between overall survival and EBV positivity through EBER-ISH, which is consistent with results obtained in other populations [49,50,51,52,53,54,55,56,57,58,59]. However, a meta-analysis conducted by Camargo et al. showed that EBV in gastric tumors is associated with early tumor stage and lower mortality, which could translate into a likely protective effect of EBV in the development of stomach carcinogenesis [51]. The genetic differences between our population and other previously investigated populations, as well as the prevalence of various EBV variants infecting our population, may help to explain the variations in these results.

EBER-ISH has been frequently used as a reference method to detect EBV due to its capability to determine virus location in the tissue [60]. However, the sensitivity of EBER-ISH has been brought into question due to a high percentage of false negatives compared to other molecular methods, such as PCR assays [60,61,62,63]. The chosen target itself could explain the false negatives of the EBER-ISH technique. EBER (EBER1 and EBER2) are non-coding RNAs expressed in latently EBV-infected cells and have an important role in malignant phenotype development by decreasing apoptosis [64], increasing interleukin expression [65], and modifying antiviral innate immunity [66]. Unfortunately, these non-coding RNAs are prone to degradation in fresh tissue samples [67], which could explain the larger number of false negative results. EBNA3A gene amplification through PCR was used to evaluate EBV presence in our study. EBNA3A belongs to the subgroup of latent viral proteins, which are essential for initiating growth transformation and maintaining latency in infected cells [68]. The advantage of using EBNA3A PCR amplification rather than the EBER-ISH method is the sensitivity, specificity, and low amount of DNA needed to detect the EBV viral load in clinical samples [69]. More importantly, EBNA3A has a sufficient phylogenetic signal to classify EBV into type 1 and type 2 [22]. In our cohort, not all the samples positive for EBV in the PCR assay concorded with the EBER-ISH assay. This may be due to the EBER RNA degradation in FFPE tissue samples. Furthermore, the probes used to detect EBER may not be able to determine all EBV variants. On the other hand, most EBER-ISH-positive samples showed results consistent with the PCR approach. The exception is represented by a single sample that did not exhibit amplification in PCR analysis; however, the presence of EBER was evident in its corresponding FFPE tissue. This may be due to the low amount of viral DNA (low copy number) along with a high copy number of EBER in the tissue, which was detectable through ISH. We propose using both techniques as complementary analyses in GC classification.

The phylogenetic reconstruction showed that EBV type-1 and type-2 were present in EBV-positive GC cases from southern Chile. EBV-1 was identified in 75% of EBV-positive samples, while EBV-2 was identified in 25%. The frequency of EBV-1 found in our study is in agreement with global studies [70,71]. The advantage of our study is the unique 12 EBV isolates sequenced from Chilean GC cases, which allows analysis of the viral genetic diversity. Access to EBV sequences enables a better understanding of virus variability and exploration of the biological properties of each viral subtype. While both EBV subtypes can infect humans [5], EBV-1 is known to efficiently immortalize B lymphocytes in vitro compared with EBV-2 [72]. In addition, EBV-2 can spontaneously enter the lytic cycle compared to EBV-1 [73]. We found EBV-1 isolates associated with three main populations in three sub-clades: Brazil–Kenya, Australia–USA, and Argentina–Brazil. Most of the isolates related to EBV were retrieved from cancer-related diseases. Unexpectedly, we detected the presence of EBV-2. Thus far, only one isolate of EBV-2 (AG876-GC1) has been associated with GC. Our study now describes three additional isolates of EBV-2 associated with GC, all of which were identified on the American continent. EBV-2 is a more frequent variant in sub-Saharan Africa and Papua New Guinea but less common in Western populations [74]. EBV-2 has also been reported as frequent in immunocompromised people [75]. In addition, the Chilean EBV isolates showed a well-defined genetic diversity among dataset isolates within each EBV-1 or EBV-2 classification. The EBV-2 cluster showed a higher geographical association with isolates from Africa and Oceania. The reason why EBV-2 has been more strongly associated with Burkitt lymphoma than GC in the African population is still unclear and constitutes another interesting topic to be studied. We hypothesize that the introduction of these EBV isolates is due to recent migrations to Chile or recent migrations of Chilean individuals abroad, where they came in contact with people infected with these viral lineages.

In conclusion, our study provides important information for an epidemiological follow-up of EBV subtypes that could be involved in GC development in southern Chile, one of the areas with the highest incidence and mortality rates of this cancer worldwide. We demonstrated a high correlation between EBER-ISH and PCR methods, suggesting that EBV diagnosis using PCR could be included to classify the different molecular subtypes of GC, as proposed for other malignancies such as lymphomas [40]. The specific molecular characteristic of EBVaGC is well established; therefore, molecular diagnosis of EBV and its genotypes would be necessary for further treatment.

## 4. Materials and Methods

### 4.1. Patients, Sample Collection, and Ethics Approval

The GC tissues used in this study were obtained from 54 male and female adult patients between 2008 and 2012. These tissues were collected immediately after surgical resection in the Hernan Henríquez Aravena Hospital, Temuco, La Araucanía, Chile. The patients provided informed consent before the collection of the samples. Two methods were used to collect the tissues: fresh-frozen and formalin-fixed paraffin-embedded (FFPE) tissues. The Universidad Autónoma de Chile Ethics Committee approved using these samples with Certificates N° 106-18 and N° 33-19. The patients’ ancestry was determined based on their surnames.

### 4.2. Determination of EBV through EBER-ISH Analysis

RNA nuclear Epstein–Barr encoding region (EBER) in situ hybridization (ISH) analysis was performed in FFPE GC tissues. Each FFPE GC tissue was cut into 4 μm thick sections to be de-waxed in xylene and rehydrated through graded concentrations of ethanol before performing the assay using Epstein–Barr Virus (EBER) PNA Probe/Fluorescein (Agilent, Santa Clara, CA, USA, Cat. Y5200). In EBER-ISH analysis, tissue samples were pre-treated with 300 μL of diluted Proteinase K for 30 min at 29 °C. Then, samples were incubated with fluorescein-labeled peptide nucleic acid (PNA) probes for 1.5 h at 55 °C and washed with distilled water, stringent wash solution, and TBS buffer, respectively. Finally, alkaline phosphatase-conjugated F(ab’) fragments of rabbit anti-FITC and TBS buffer were added to the samples. The Dako PNA Detection ISH commercial kit (Agilent, USA, Cat. K5201) was used for detection. Finally, slides were contrasted with Kernechtrot staining.

The slides were examined independently by two pathologists using a semiquantitative scoring system, in which staining intensity was scored as 0 (absent), 1 (mild), 2 (moderate), and 3 (strong). For statistical analysis, the sum score of intensity and extent of staining was grouped into negative/low (final score of 0) or positive/high (final score of 1 to 2; Table 1). The percentage of positive cells was quantified as 0 (none), 1 (1–25%), 2 (26–50%), 3 (51–75%), and 4 (76–100%).

### 4.3. DNA Extraction, PCR Amplification, and Sequencing

DNA extraction from fresh-frozen GC tissues was carried out using a commercial kit (E.Z.N.A Tissue DNA Kit; Omega Bio-Tek, Norcross, GA, USA) according to the manufacturer’s instructions. EBV positivity was assessed through PCR amplification using the following primers for the N-terminal region (840 pb) of the EBNA3A gene: forward (EBNA3A2F) 5′CGTTRMGGGCTAGTATGGGC3′ and reverse (EBNA3A2R) 5′ACCCACTGTAATACGCCCG3′. EBV-positive samples were subjected to a complementary round of PCR, amplifying ~1000 bp more from the N-terminal region of the EBNA3A gene, using the following primers: forward (EBNA3A1F) 5′CACAKGCTTGGAATGCCGGCTT3′ and reverse (EBNA3A1R) 5′GCCCATACTAGCCCKYAACG3′. Cycling conditions were 3 min at 98 °C, 35 cycles of 30 s at 95 °C, and 30 s at 55 °C, followed by 1 min at 72 °C.

The EBNA3A amplification products of EBV-positive GC samples obtained in this study were sequenced using the Sanger methodology on the Macrogen platform (https://dna.macrogen.com/, accessed on 3 January 2022).

### 4.4. Sequence Datasets and Alignments

A collection of 188 EBV sequences of EBNA3A (Supplementary Material, Table S1) were retrieved from the NCBI database (https://www.ncbi.nlm.nih.gov, last accessed on 6 June 2022) and analyzed conjointly with the EBVaGC samples sequenced in this study. Multiple sequence alignment of the EBNA3A coding region (CDS) was performed using MAFFT (http://mafft.cbrc.jp/alignment/software/, accessed on 18 July 2022), and the alignment was manually inspected using Bioedit 7.2.5 [76]. Chilean sequences have been assigned the accession numbers MN842146-MN842157 in NCBI.

### 4.5. Phylogenetic Analyses

A maximum-likelihood (ML) tree was inferred based on the EBNA3A alignment using PhyML (http://www.atgc-montpellier.fr/phyml/, accessed on 12 September 2022) with 1000 bootstrap replicates, and the most appropriate nucleotide substitution model was selected using the SMS program [77] based on the Akaike information criterion (AIC). The best-fitting model selected was the GTR + T + I. Figtree 1.4.4 software (http://tree.bio.ed.ac.uk/software/figtree/, accessed on 21 November 2022) was used to visualize the tree.

### 4.6. Statistical Analysis

All statistical analyses were performed using GraphPad Prism 9.0 software (Prism GraphPad, San Diego, CA, USA). EBER staining, EBV positivity, and the patients’ clinicopathological features were analyzed using the chi-square or Fisher’s exact test. Survival analyses were performed using log-rank tests. In all these cases, a *p*-value < 0.05 was considered statistically significant.

## 5. Conclusions

In conclusion, our study highlights a high prevalence of EBVaGC (22%) among patients in southern Chile, with a significant association with diffuse-type histology and a tendency to be more frequent in men than women. EBVaGC was not shown to have a better prognosis than EBV-negative GC. We demonstrated that PCR could be used to identify a higher number of positives compared to the standard methodology. Our phylogenetic analyses confirmed the presence of EBV-1 and EBV-2 in southern Chile and the genetic diversity among the isolates.

Our study provides important insights that can guide the epidemiological monitoring of EBV subtypes (EBV-1 and EBV-2) that may be involved in GC development in southern Chile. In light of the finding that EBV-2 has been more commonly associated with Burkitt lymphoma than GC in people from Africa and Oceania, further research is needed to explore these strains’ potential involvement in GC development. To this end, we recommended the incorporation of PCR-based EBV diagnosis to avoid false negative results, particularly in geographic areas where EBV-associated neoplasms are prevalent.

## Figures and Tables

**Table 1:** Interpretation of EBER-ISH scores.

Staining Intensity	Cell Percentage	Score Sum	Final Score		Expression Level
0 Absent 1 Mild 2 Moderate 3 Strong	0 0% 1 1–25% 2 26–50% 3 51–75% 4 76–100%	0–2	0	Negative/Low	Absent/0% cells Mild/1–25% cells
		3–4 5–7	1 2	Positive/High	Mild/26–100% cells Moderate/1–100% cells Strong/1–100% cells

**Table 2:** EBV frequency in gastric carcinomas according to different clinicopathological features.

	Total % (*n*)	EBV+ % (*n*)	EBV− % (*n*)	*p*
Age				0.220
>65 years old	55.5% (30)	41.7% (5)	59.5% (25)	
<65 years old	44.5% (24)	58.3% (7)	40.5% (17)	
Gender				0.060
Female	29.6% (16)	8.3% (1)	35.7% (15)	
Male	70.4% (38)	91.7% (11)	64.3% (27)	
Histological diagnosis				0.003 *
Diffuse	37% (20)	75% (9)	26.2% (11)	
Intestinal	63% (34)	25% (3)	73.8% (31)	
Bormann classification				0.550
I–II	39.2% (20)	41.7% (5)	38.5% (15)	
III–V	60.8% (31)	58.3% (7)	61.5% (24)	
Anatomical classification				0.418
Cardia	74.5% (35)	81.8% (9)	72.2% (26)	
No cardia	25.5% (12)	18.2% (2)	27.8% (10)	
Infiltration				0.384
pT0	1.9% (1)	0% (0)	2.4% (1)	
pT1	7.5% (4)	0% (0)	9.8% (4)	
pT2	13.2% (7)	25% (3)	9.8% (4)	
pT3	67.9% (36)	58.3% (7)	70.7% (29)	
pT4	9.4% (5)	16.7% (2)	7.3% (3)	
Lymph nodes				0.075
pN0	35.8% (19)	50% (6)	31.7% (13)	
pN1	11.3% (6)	25% (3)	7.3% (3)	
pN2	20.8% (11)	0% (0)	26.8% (11)	
pN3	32.1% (17)	25% (3)	34.1% (14)	
TNM stage				0.256
0	1.9% (1)	0% (0)	2.4% (1)	
I	13.2% (7)	16.7% (2)	12.2% (5)	
II	35.8% (19)	50% (6)	31.7% (13)	
III	41.5% (22)	16.7% (2)	48.8% (20)	
IV	7.5% (4)	16.7% (2)	4.9% (2)	
Differentiation				0.196
Poor	61.5% (32)	83.3% (10)	55% (22)	
Moderate	34.6% (18)	16.6% (2)	40% (16)	
Good	3.9% (2)	0% (0)	5% (2)	

EBV: Epstein–Barr virus; (+): positive; (−): negative. * It represents that there are statistically significant differences.

## Data Availability

Sequences obtained for the *EBNA3A* gene from Chilean patients are available in the NCBI database. The assigned accession numbers are MN842146-MN842157 (https://www.ncbi.nlm.nih.gov/nuccore/MN842146.1/, last accessed on 18 April 2022).

## Supplementary Materials

The supporting information can be downloaded at: https://www.mdpi.com/article/10.3390/ijms241411276/s1.
