# Supplementary material for: Exploring the Genetic Diversity of Epstein–Barr Virus among Patients with Gastric Cancer in Southern Chile
Source: Int J Mol Sci. 2023 Jul 10;24(14):11276. doi: 10.3390/ijms241411276 (PMC10378801; doi:10.3390/ijms241411276)
Supplement: Supplementary file 1 [file ijms-24-11276-s001.zip › ijms-2434249-SI.pdf]

Supplementary Table S1 List of genome sequences retrieved from NCBI database used in this investigation

Isolate	Accession number
NC_7605	B95.8Raji
NC_9334	AG876
AP015016	YCCEL1
KF373730	M81
LN827556	Cheptages
LN827576	sLCL-IS1.20
LN827580	sLCL-2.16
LN827596	sLCL-IM1.02
LN827560	sLCL-2.14
LN827587	sLCL-2.21
LN827578	sLCL-IS1.13
LN827800	Jijoy
LN827575	sLCL-IS1.14
LN827591	sLCL-2.15
LN827590	sLCL-IM1.05
LN831023	sLCL-2.22
LN827545	Daudi
LN827548	P3HR1c16
LN827567	sLCL-IM1.09
LN827583	sLCL-IM1.17
LN827589	sLCL-IS2.01
LN824209	HKN14
LN827588	SLCL-IS1.19
LN827524	HL11
LN827544	Wewak1
LN827557	Bl36
LN827547	HKN15
LN824224	HKN19
LN827570	sLCL-IS1.01
LN827569	sLCL-IS1.11
LN827592	sLCL-IS1.10
LN827572	sLCL-IS1.18
LN827593	sLCL-IS1.12
KT273942	EBVaGC1
LN827527	M-ABA
LN827564	HL04
LN824205	sLCL-1.12
LN827594	sLCL-IS1.0.7
LN827522	HL09
LN827549	D3201.2
LN827561	YCCEL1
LN827582	sLCL-BL1.03
LN827526	Bl37
LN827562	sLCL-1.19
LN827586	sLCL-IS1.15
LN827525	C666-1-reseq
LN827581	sLCL-IM1.05
LN827550	sLCL-1.11
LN827563	sLCL-1.18
LN827566	sLCL-1.06
LN827571	sLCL-BL1.20
LN827585	sLCL-1.04
LN827799	sLCL-IM1.16
LN827577	sLCL-1.17
LN827552	sLCL-1.08
LN827568	sLCL-1.24
LN827546	HL02
LN824225	HL08
LN827573	sLCL-1.10
LN827739	LCLB95-8-del-EBER2-reseq
LN827565	sLCL-1.07
KR063344	RPF
LN827558	sLCL-1.02
LN827553	sLCL-IS1.08
KT273949	EBVaGC9
LN827559	pLCL-TRL-595
KC440852	K4413-Mi
LN824203	Mak1-duplicate
LN827584	sLCL-IS1.06
KR063345	FNR
KR063342	H03753A
KP968264	H002213
AJ507799	B95.8Raji
KP968263	H058015C
KR063343	CV-ARG
KP968259	SCL
LN827555	X50-7
KT001103	SG
KP968261	HU11393
LN824226	HL01
KP968262	H018436D
KC440851	K4123-Mi
LN827579	sLCL-1.13
LN827595	sLCL-IS1.03
LN824204	HL05
LC150338	HNNPC5
LN827574	sLCL-1.09
KP968257	CCH
LN827597	sLCL-IS1.04
KT273943	EBVaGC2
KT273945	EBVaGC5
LN824208	Akata-reseq
KT823508	LC3

LN824142	Saliva1
LN827551	Makau
LN824206	pLCL-TRL-post
KP968258	MP
KC207814	Mutu
KT273947	EBVaGC7
KP968260	VGO
KT273948	EBVaGC8
KT273944	EBVaGC4
AY961628	GD1
KT823507	LC2
LC149491	HNNPC2
KT254013	EBVaGC3
KT273946	EBVaGC6
KT823509	LC4
LN827523	L591
LN824207	pLCL-TRL1-pre
KT823506	LC1
KF992565	HKNPC3
KF992568	HKNPC6
JQ009376	HKNPC1
LC150741	HNNPC6
LC150337	HNNPC4
KF992570	HKNPC8
LC137018	HNNPC1
KF992567	HKNPC5
KC207813	Akata
KC617875	C666-1
KF992566	HKNPC4
LC150742	HNNPC7
LC150327	HNNPC3
KJ411974	C666-1
KF992571	HKNPC9
KP735248	GC1
KF992569	HKNPC7
KX125052	SNU-719
KF992564	HKNPC2
LC150743	HNNPC8
KX125050	GC-EBV1
AP015015	SNU719
KX125053	YCCEL1
KF717093	Raji
HQ020558	GD2
MF547453	E1583_BCv1
MF547454	E1583_OWv1
MF547455	E1583_BCv7
MF547456	E1583_OWv7
MF547457	E1587_BCv1
MF547458	E1587_OWv1

MF547460	E1587_OWv7
MF547461	E1536_BCv1
MF547462	E1536_OWv1
MF547463	E1563_BCv1
MF547464	E1536_OWv7
MF547465	E1536_BCv7
MF547466	E1548_BCv1
MF547467	E1548_OWv1
MF547468	E1548_BCv7
MF547469	E1548_OWv7
MF547470	E1563_OWv1
MF547471	E1563_OWv7
MF547472	E1563_BCV7
MF547473	E1590_BCv1
MF547474	E1590_OWv1
MF547475	E1590_BCv7
MF547476	E1590_OWv7
MF547477	E1492_BCv1
MF547478	E1492_OWv1
MF547479	E1492_BCv7
MF547480	E1492_OWv7
MF547481	E1503_BCv1
MF547482	E1503_OWv1
MF547483	E1503_BCv7
MF547484	E1503_OWv7
MF547485	E1578_BCv1
MF547486	E1578_BCv7
MF547487	E1578_OWv7
MF547488	E1578_OWv1
MF547489	E1577_BCv1
MF547490	E1577_OWv1
MF547491	E1577_OWv7
MF547492	E1577_BCv7
MG021314	EBVaGC8-2
MG021305	YCCEL1-GC1
MG021315	EBVaGC8-3
MG021317	EBVaGC5-1
MG021308	Mutu-GC1
MG021307	Akata-GC1
MG021312	Akata-GC1
MG021316	HKNPC6-GC1
MG021310	Mutu-GC3
MG021311	Mutu-GC4
MG021309	Mutu-GC2
MG021313	EBVaGC8-1
MG021306	YCCEL1-GC2
MN842146	TCO_CL_V29
MN842147	TCO_CL_V32
MN842148	TCO_CL_V33

MN842149	TCO_CL_V34
MN842150	TCO_CL_V39
MN842151	TCO_CL_V47
MN842152	TCO_CL_V53
MN842153	TCO_CL_V55
MN842154	TCO_CL_V62
MN842155	TCO_CL_66
MN842156	TCO_CL_127
MN842157	TCO_CL_130
